# News and Notes

**Published:** 1904-02

**Authors:** 


### *NEWS AND NOTES.*

DR. WILLIAM OSLER has been elected president of the New Sydenham Society of London.

THE American Röntgen Ray Society held its annual convention in Philadelphia on December 10 and 11.

IT is announced from Copenhagen that Dr. Finsen of that city, to whom the Nobel medical prize was awarded recently, is seriously ill.

DR. M. ALLEN STARR has been elected a corresponding member of the Neurological Society of the United Kingdom, London, in recognition of his contributions to the study of nervous diseases.

THE Iowa Society for the Suppression of Disease and Degeneracy has prepared a bill, it is said, to be introduced in the coming assembly, providing for a physician's certificate of examination before a marriage license can be issued.

SMALLPOX as officially reported in the United States for the period from June 27, 1903, to December 4, 1903, amounts to 11,908 cases, with 491 deaths, as against 14,262 cases, with 729 deaths, in the corresponding period of last year.

JUDGE CLEVELAND, of New Haven, recently refused to appoint an Eddyite as guardian to the nine-year-old daughter of Isaac W. Stiles, of New Haven. The judge made this decision after asking some questions as to prospective conduct in case of the serious sickness of the child.

ON invitation of Dr. John M. Fisher, president of the Philadelphia Obstetrical Society, Dr. William R. Pryor, of New York, delivered an address on "Puerperal Sepsis" before that body. He was afterward entertained at a dinner, and a reception and smoker were given in his honor.

---

A GERMAN professor, says *American Medicine*, has invented a process of silver-plating dead bodies so as to convert them into metallic images of the individuals as they were when in life. Gold plate can be used if the relatives can afford it. But as the expense of silver-plating a body is $12,500, there are probably few relatives who would deem themselves justified in squandering the deceased's estate on such a memorial.

---

AT the last meeting of the Berlin Society for Combating Venereal Disease, Professor Kuttner read a paper on quacks and venereal diseases. Enlarging on the well-known fact that it is for the syphilitic patient above all others that quacks spread their nets, he quoted official statistics to prove that of Berlin female quacks 14.4 per cent., and of Berlin male quacks no less than 29 per cent., are individuals previously convicted of crime.

---

JOHN A. TURNER, an Eddyite, died November 29, at Savannah, Ga., while under "science" treatment. The health officer refused to issue a burial certificate, and called on the coroner to investigate the death. The coroner reported that Turner died from the rupture of a blood-vessel, and that he was not attended by a physician. The case will be laid before the grand jury and indictments asked on two grounds: First, that the Eddyites who attended Turner violated the law requiring licenses; and secondly, that there was criminal negligence in permitting the man to suffer without proper attention.

PRESIDENT HARPER of the University of Chicago, says *American Medicine,* has warned the students of a typhoid-fever epidemic at the university. Several cases have developed recently, and the authorities, fearing a serious siege, have begun vigorous efforts to stamp out the disease before it gets a firmer hold among the students. Before a special meeting of all the students Mr. Harper personally asked the university men and women to do all in their power to help the authorities in their efforts to rid the university of the disease before the classes have to be broken up and the students sent to their homes. The students were asked to leave boarding-places where the water was not boiled, and to take other precautions.

---

THAT the sense of smell is greatly impaired in old age appears from observation by a French physiologist, M. Vaschide, who communicates his results to the Academy of Science, Paris. To quote a report in *Cosmos* (Oct. 31): "Old people seem to have an atrophied sense of smell, and it is a remarkable fact that they do not seem to be conscious of this infirmity. Even when they can scarcely distinguish one familiar odor in ten, and when they take strong-smelling liquids for pure water, they assert that they enjoy the perfume of flowers. Their visual images make up for the absence of olfactive images, for they recognize the perfumes of the flowers when they are able to see them. The olfactive image thus has an independent intellectual existence, since it is capable of functional revival." (Translation made for the *Literary Digest.*)

---

THE Royal Commission on Arsenical Poisoning from Food and Drink of Great Britian, says *American Medicine,* has recommended the prohibition of the sale of beer and other liquid food, or any liquid entering into the composition of food, which contains 1/100 grain or more of arsenic per gallon, and also the prohibition of the sale of solid food containing 1/100 grain per pound. The commissioners state there are serious defects in the present methods to safeguard the public, and urge that more extended powers be given to the authorities to condemn unwholesome food. They recommended the establishment of official standards at the creation of a board of reference, to which could be referred specific points. Their decisions should be carried out by the department concerned, and the latter's action should be subject to the control of Parliament.

---

PRETTY CHRISTMAS PARTY TO EMPLOYEES.—One of the pleasantest events of the holiday season in the drug circles was a buffet luncheon given on last Thursday by Fairchild Bros. & Foster to their employees. It was a complete surprise to the guests, who numbered about 140. A big table in the work-room on the fourth floor, at 74 Laight street, fairly groaned with all the good things prepared by Maresi's chefs, and was decorated with potted plants presented to members of the firm by the members of the office staff.

B. T. Fairchild welcomed the guests to the "Christmas party," as it was called, and E. W. Dusenberry responded, after which Messrs. S. W. Fairchild and M. G. Foster also spoke. There was music during and after the luncheon, which began at 12:30, and at three o'clock the annual presents in gold were distributed among the employees, and the rest of the day was declared a holiday.

---

THE Warren Triennial Prize (founded by the late Dr. J. Mason Warren in memory of his father) of $500 will be awarded in 1904 for the best dissertation on some subject in physiology, surgery, or pathological anatomy, the arbitrators being the physicians and surgeons of the Massachusetts General Hospital. A high value will be placed on original work. Competing essays must be legibly written, and suitably bound, so as to be easily handled. The name of the writer must be inclosed in a sealed envelop, on which must be written a motto corresponding with one on the accompanying essay. Any clew given by a dissertation or by a writer which reveals his name before the award of the prize will disqualify him. If no essay is considered worthy, no award will be made. April 14, 1904, is the last day on which manuscripts may be received by Dr. Herbert B. Howard, resident physician, Massachusetts General Hospital, Boston, Mass.

THE bulletin of the Chicago Health Department for the week ended November 21 says: "In 1855 began the elevation of the city level so as to secure drainage and sewerage, and in 1856 the first sewer-pipe was laid., During the intervening period—that is, between 1851 and the close of last year—the citizens of Chicago have paid more than $100,000,000 for these two essential 'sanitary conditions,' namely, a water-supply system of more than 11,000,000 linear feet, and a drainage and sewerage system of nearly 9,000,000 feet. This does not include the cost of lifting the city bodily to a height of eight feet above the original level of the site— a necessity for the drainage system. During the quarter of a century preceding water-supply and sewerage the average annual death-rate was 33.87 per 1000. In the succeeding twenty-five years this rate had fallen to 22.37 per 1000, and in the subsequent period, ended in 1902, it still further fell to 18.16 per 1000, a little more than one-half the rate of the first twenty-five years of the city's existence."

---

THE thirtieth annual meeting of the Mississippi Valley Medical Association will be held at Cincinnati, Ohio, October 11, 12 and 13, 1904. Dr. B. Merrill Ricketts has been elected chairman of the Committee of Arrangements. The following are the officers of the Association elected at Memphis: President, Edwin Walker, M.D., Evansville, Ind.; President-elect, Hugh T. Patrick, M.D., Chicago, Ill.; First Vice-President, Bransford Lewis, M.D., St. Louis, Mo.; Second Vice-President, George W. Cale, Jr., M.D., Springfield, Mo.; Secretary, Henry Enos Tuley, M.D., Louisville, Ky.; Assistant Secretary, S. C. Stanton, M.D., Chicago, Ill.; Treasurer, Thomas Hunt Stucky, M.D., Louisville, Ky. The following resolution was offered by Dr. S. P. Collins, of Hot Springs, Ark., at the Memphis meeting:

WHEREAS, The value of perfect sight and hearing is not fully appreciated by educators, and neglect of the delicate organs of vision and hearing often leads to disease of these structures; therefore, be it

*Resolved*, That it is the sense of the Mississippi Valley Medical Association that measures be taken by boards of health, boards of education, and school authorities, and, where possible, legislation secured, looking to the examination of the eyes of all school-children, that disease in its incipiency may be discovered and corrected.

